# A novel mechanism by which ACTA2-AS1 promotes cervical cancer progression: acting as a ceRNA of miR-143-3p to regulate SMAD3 expression

**DOI:** 10.1186/s12935-020-01471-w

**Published:** 2020-08-05

**Authors:** Lingli Luo, Min Wang, Xianping Li, Can Luo, Shan Tan, Sheng Yin, Lei Liu, Xiaolin Zhu

**Affiliations:** Department of Laboratory Medicine, The Second Xiangya Hospital, Central South University, Changsha, 410011 Hunan China

**Keywords:** Cervical cancer, ACTA2-AS1, miR-143-3p, SMAD3, ceRNA

## Abstract

**Background:**

Long non-coding RNAs (LncRNAs) have been increasingly confirmed to be abnormally expressed in human cancer and closely related to tumorigenesis. LncRNA ACTA2-AS1 is abnormally expressed in multiple tumors and participates in their development. However, whether ACTA2-AS1 plays a role in the development of cervical cancer (CC) and the exact mechanism of its role has not been elucidated.

**Methods:**

Quantitative real-time PCR (qRT-PCR) was conducted to detect the expression level of messenger RNA of ACTA2-AS1, miR-143-3p and SMAD3 in tumor tissues and cells. Additionally, SMAD3 protein expression by western blots in cells. Small interference RNA against ACTA2‐AS1 or SMAD3 and miR‐143‐3p mimic/inhibitor was designed and transfected into CC cell lines to investigate their correlations and potential impacts on cell function. Cell Counting Kit-8 (CCK-8) assay, colony formation, cell cycle assay, transwell assay and flow cytometry analysis were performed to detect the specific effects on cell line proliferation, metastasis and apoptosis.

**Results:**

ACTA2-AS1 was significantly increased in CC tissues and cells and miR‐143‐3p was down-regulated. Clinically, the higher expression of ACTA2-AS1 was significantly correlated with higher FIGO stage. Loss-of-function assay revealed that silencing of ACTA2-AS1 inhibited cell proliferation, colony formation, migration and promoted apoptosis in CC. Additionally, Pearson correlation analysis showed that the expression of ACTA2-AS1 and miR-143-3p were negatively correlated. Dual-luciferase reporter assay and further mechanistic experiments confirmed that ACTA2-AS1 could sponge and regulate the expression of miR-143-3p. Furthermore, SMAD3 was the target gene of miR-143-3p and ACTA2-AS1 could upregulate SMAD3 through acting as a competitive endogenous RNA (ceRNA) of miR-143-3p. Finally, rescue assay demonstrated that the ACTA2-AS1/miR-143-3p/SMAD3 axis played an important role in the proliferation, migration and apoptosis of CC cells.

**Conclusions:**

In summary, our study revealed that ACTA2-AS1 upregulates SMAD3 by competitively binding miR-143-3p, thereby accelerating CC progression. The ACTA2-AS1/miR-143-3p/SMAD3 axis can play a crucial role in cervical carcinogenesis, providing new clues for the early diagnosis and treatment of CC.

## Background

Cervical cancer (CC) is a common gynecological malignant tumor, accompanied by a trend of rejuvenation in recent years [1]. CC continues to be the second leading cause of cancer death in women aged 20 to 39 years, causing 10 premature deaths per week in this age group [2]. The present treatment for CC is mainly based on surgery, radiation, chemotherapy sequentially or concomitantly. Nevertheless, cancer survival of uterine cervix has not improved since the mid‐1970s. The unsatisfactory survival rates of CC largely reflected a lack of major treatment advances for patients with recurrent and metastatic disease [3, 4]. For women who have the opportunity of having their disease early detected by early screening, they could be cured with appropriate treatment to avoid their progression to invasive cancer [5]. Therefore, the investigation of the molecular mechanisms of tumorigenesis and progression remains crucial for the early diagnosis and timely treatment of CC.

It is known that long non-coding RNAs (lncRNAs) modulates the development of many human cancers [6]. LncRNAs can affect various aspects of cellular homeostasis, including cell proliferation, migration, apoptosis or genomic stability [7]. According to previous studies, some lncRNAs could play a crucial role in the progression of CC, such as GHET1 [8], SNHG7 [9] and WT1-AS [10]. In our previous RNA-sequencing analysis (unpublished data), we found that lncRNA ACTA2 antisense RNA 1 (ACTA2-AS1) showed significant higher expression in CC tissues compared to adjacent normal tissues (ANT). ACTA2-AS1, also named as ZXF1, UC001kfo, and uc001kfo.1 (Gene id: 100132116), is a lncRNA consisting of five exons, located at GRCh38, 10q23.31. Recent studies revealed that ACTA2-AS1 was correlated with the development of several cancers such as liver cancer [11], lung adenocarcinoma [12], hepatocellular carcinoma [13] and breast cancer [14]. However, whether ACTA2-AS1 plays a role in the development of CC and the exact mechanism of its role remains unclear. Since our previous study found that ACTA2-AS1 was up-regulated in CC, we hypothesized that ACTA2-AS1 may be a participant in the process of CC. Therefore, this study firstly aimed to explore the expression level and its specific function of ACTA2-AS1 in CC growth.

It is widely acknowledged that lncRNAs can regulate the expressions of microRNAs (miRNAs) through a competitive endogenous RNA (ceRNA) regulatory network [15]. MiRNAs mainly regulates the expression of protein-coding genes through post-transcriptional patterns [16, 17]. Thus, lncRNAs could competitively bind with miRNA-response elements to upregulate down-stream mRNAs. However, whether ACTA2-AS1 could act as a ceRNA to regulate expressions of down-stream miRNAs in CC is still unknown. In our previous research, lncRNA-miRNA co-expression analysis showed that ACTA2-AS1 was significantly correlated with miR-143-3p and miR-143-3p was down-regulated in CC. As a widely researched cancer related-miRNA, miR-143-3p has been verified to be lowly expressed in various cancers and involved in the pathogenesis of cancers. Meanwhile, miR-143-3p could be regulated by multiple lncRNAs through ceRNA mechanism, such as MALAT1 in Hepatocellular Carcinoma [18] and SARCC in Renal Cell Carcinoma [19]. On this basis, we speculated that ACTA2-AS1 may act as a ceRNA of miR-143-3p to regulate its targeted mRNA, which in turn promotes tumorigenesis in CC.

Here, we explored the effects of lncRNA ACTA2-AS1 in CC and found that ACTA2-AS1 promoted cell proliferation and migration and inhibited apoptosis of CC cells. Further mechanism analysis revealed that ACTA2-AS1 regulated SMAD3 expression to accelerate progression of CC by sponging miR-143-3p. Collectively, this study is proposed to elucidate the function role and molecular mechanism of ACTA2-AS1 in CC tumorigenesis and provide a novel promising therapeutic target.

## Methods

### Patients and materials

Three paired CC tissues and ANT were obtained for RNA sequencing. In addition, another total of 54 paired cervical carcinoma tissues and ANT were obtained from patients at our hospital from 2016 January to 2019 January. All patients had not received radiotherapy or chemotherapy preoperatively and the samples of patients with other major diseases were excluded. Based on a diagnostic selection sample for cervical cancer, the diagnosis is determined by at least two pathologists. The clinical features of patients in our study were obtained from medical records. The clinical staging and clinicopathological classifications were determined based on the International Federation of Obstetrics and Gynecology (FIGO). Tissues were collected at surgery and snap-frozen immediately in liquid nitrogen until needed. Informed written consent was obtained from each patient before surgery. This study was approved by the Ethics Committee of the Second Xiangya Hospital, Central South University. All patients provided with written informed consent.

### Cell culture and cell transfection

Human Cervical cancer cell lines (HeLa, SiHa and CaSki) and human normal cervical epithelial cell line (HcerEpic) were all obtained from Cell Bank of the Chinese Academy of Science (Shanghai, China). All cells were incubated in Dulbecco’s Modified Eagle Medium (DMEM) (Gibco, Carlsbad, CA) containing 10% FBS (Invitrogen) and 1% penicillin–streptomycin (10,000 U/ml; Invitrogen, Carlsbad, CA). Cells were maintained at 37 °C and 5% CO_2_ in a humidified incubator for 4–6 h before changing to complete medium. For ACTA2-AS1 up-regulation, pcDNA3.1 vector (Genechem Company, Shanghai, China) were subcloned with ACTA2-AS1 (pcDNA-ACTA2-AS1). Besides, small interference RNA against ACTA2‐AS1 (si‐ACTA2‐AS1#1/2/3), small interference RNA against SMAD3 (si‐SMAD3#1/2/3), miR‐143‐3p mimics, miR‐143‐3p inhibitors, and the corresponding negative control (NC) were designed and synthesized by GenePharma (Shanghai, China). Sequences of these transfections were listed in Additional file 1: Table S1. Cell transfection was conducted by using Lipofectamine 3000 kit (Thermo Fisher Scientific, Inc.). After 24–48 h of transfection, further experiments were carried out based on the purpose of the experiment. All assays were repeated at least three times.

### Quantitative real-time PCR (qRT-PCR)

Under the protocol of the manufacturer, TRIzol reagent ((Thermo Fisher Scientific, Inc.) was used to extract RNA from cervical cells and tissues. The concentration and quality of RNA were detected using NanoDrop 2000 Spectrophotometer (Thermo Fisher Scientific, Wilmington, DE). Then mRNA and miRNA were reverse transcribed into cDNA using a PrimeScript RT Reagent Kit (Takara, Dalian, China). Furthermore, qRT‐ PCR analysis was performed to detect ACTA2‐AS1 and SMAD3 expression using SYBR Green PCR Master Mix according to the manufacturer’s protocol (Applied Biosystem, Waltham, MA). The miR-143-3p expression was tested by microRNAs qPCR Kit (Sangon, Shanghai, China). GAPDH and U6 were used as an internal reference. The relative RNA levels of ACTA2-AS1, miR143-3p, and SMAD3 were determined by using the formula of 2^−ΔΔCt^. The primers in our experiment are as follows: ACTA2-AS1-F: 5′-GTTCTGGAGGCTGGCTTGATATGG-3′, ACTA2-AS1-R: 5′-TCCTTCATCGGTAGGCAACAAACG-3′; SMAD3-F: 5′-CAGCCATGTCGTCCATC-3′, SMAD3-R: 5′-CTCGCACCATTTCTCCTC-3′; miR-143-3P: 5′-CGTGAGATGAAGCACTGTAGCTC-3′; GAPDH-F: 5′-CTCAGACACCATGGGGAAGGTGA-3′, GAPDH-R: 5′-ATGATCTTGAGGCTGTTGTCATA-3′; and U6: 5′-ATTGGAACGATACAGAGAAGATT3′. All assays repeated at least three times.

### Western blot

Protein was extracted from CC cells (1 × 10^7^) seeded in a six-well plate by RIPA buffer (Thermo Fisher Scientific, CA, USA) according to the manufacturer’s instructions. Then BCA Protein Assay Kit (Beyotime, Shanghai, China) was used to determine the concentration of protein. Equal amounts of protein samples were separated by 12 percent SDS-PAGE and were transferred into PVDF membranes. The membranes were blocked with skim milk for 1 h and then probed with primary antibodies anti‐Smad3 (1:1000, ab28379; Abcam, Cambridge, MA), Bax (1:5000, ab182733; Abcam, Cambridge, MA) and Bcl2 (1:5000, ab117115; Abcam, Cambridge, MA) incubated at 4 °C overnight. After washed three times with 0.1% tween‐PBS (TBST), the membranes were incubated by secondary HRP-conjugated antibody at 37 °C for an hour. GAPDH was used as a loading control. After rewashed three times by 0.1% TBST, the results were analyzed using an enhanced chemiluminescence kit (GE Healthcare, Chicago, IL) and visualized using an imaging system (Bio-Rad, CA, USA). All measurements were performed at least three times.

### Cell proliferation assay

After the required transfection, HeLa and SiHa cells were seeded in 96-well plates (1 × 10^3^ cells/well) overnight. Each group was set with 5 replicate wells. After 0, 24, 48, 72, and 96 h, 10 μl of Cell Counting Kit-8 (CCK-8) reagent was added into each well to analyze cell viability. Then the absorbance of each well was measured at OD 490 nm using a spectrophotometer (Thermo Fisher Scientific, Inc.) after incubation for 1 h at 37° C. All assays were conducted at least three times.

For colony formation assay, transfected cells were put into 6-well plates (1000 cells/well). After incubation for 10 days, the cells were washed twice by PBS (Invitrogen). The cells were fixed by formaldehyde for 20 min and stained with 1% crystal violet for 30 min. Finally, the numbers of visible colonies were imaged and counted under a microscope. All experiments were conducted in triplicates.

### Detection of apoptosis

HeLa and SiHa cells were transfected in 6-well plates according to the experimental purpose and cultured for 24 h. The cells were then digested and collected, and the cell density was adjusted to 2 × 10^5^/ml. Then the apoptosis was detected by Annexin V- FITC Apoptosis Detection Kit (KeyGEN, China) According to the instructions of manufacturer. The data was analyzed using Flow cytometry (BD, USA) and cells in the lower right quadrant were considered as apoptotic.

### Cell cycle assay

For the cell cycle assay, logarithmic growth cells of each transfection group were seeded into six-well plates at 1 × 10^5^ per well. After 24 h of culture, cells were harvested after trypsinization and washed 3 times with pre-chilled PBS. Add 3 ml of 75% ethanol to the cells and incubate overnight at 4 °C. Propidium iodide (PI) was then added and stained in the dark for 25 min, and the cell cycle of each group was then determined by flow cytometry.

### Cell migration assay

Transwell chambers (8 μm pore size, Corning) were used for cell migration assays. The transfected HeLa and SiHa cells were resuspended in 200 μl serum‐free medium at a density of 1 × 10^5^ cells/ml and then seeded in the upper chambers. A total of 600 μl medium containing 20% FBS was added to the lower chambers for inducing cell migration. After 24 h of incubation, the cells on the upper membrane surface were manually removed and the cells migrated to the lower surface were washed twice with PBS. Then they were fixed with methanol for 30 min, stained with 20% Giemsa for 30 min. The filters were washed with PBS twice and stained migrated cells were counted under a microscope. Every experiment was conducted for more than three times.

### Dual‐luciferase assay

We used LncBase Predicted v.2 [20] to predict miR-143-3p binding site in ACTA2-AS1. The putative miR-143-3p target binding sequence in ACTA2-AS1 (ACTA2‐AS1‐wt) and its binding site mutant (ACTA2‐AS1‐mut) were then designed and integrated into a psi-CHECK2 reporter vector to construct Dual-luciferase reporter plasmids. Then, HeLa and SiHa cells were co-transfected with ACTA2‐AS1‐wt or ACTA2‐AS1‐mut reporter plasmid and miR‐143‐3p mimics or miR‐NC, respectively. After transfection for 48 h, the luciferase activity was detected by Dual‐Luciferase assay system (Promega, Madison, WI). All values were given relative to appropriate transfection negative controls.

### Statistical analysis

All statistical analysis was accomplished by GraphPad Prism 5.0 (GraphPad, San Diego, CA). All data were expressed as the mean ± standard deviation (SD) of more than three independent experiments. Spearman correlation analysis was used to analyze whether there was a correlation between ACTA2-AS1 and miR-143-3p. Differences between two groups were assessed using a two-group t-test or Chi square test, and one-way ANOVA was used to analyze differences among more than two groups. When the P value was less than 0.05, it was considered statistically significant.

## Results

### ACTA2-AS1 was strongly expressed in cervical cancer tissues and cell lines

**Fig. 1:**
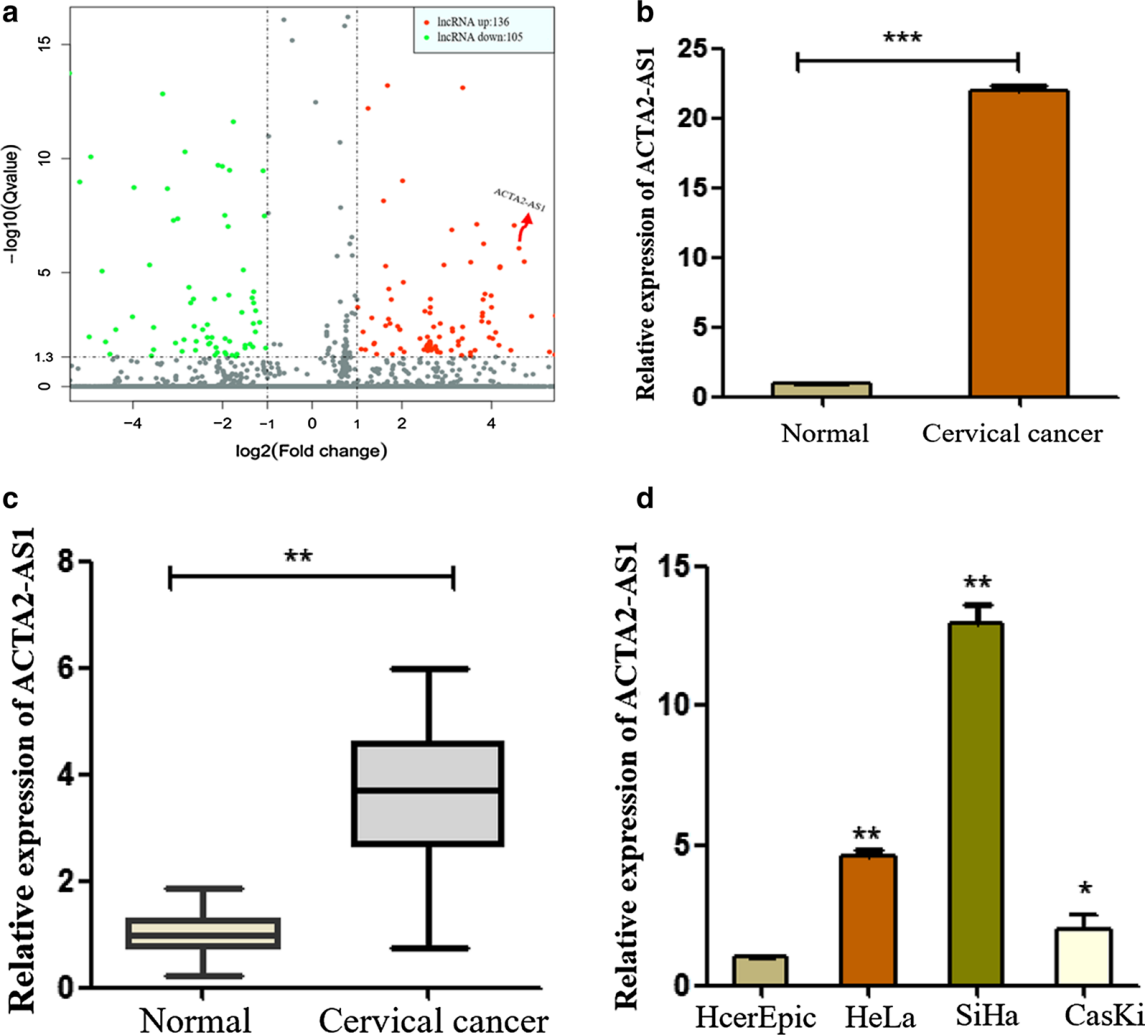
ACTA2-AS1 is up-regulated in CC tissues and cells. **a** Volcano plot of differentially expressed lncRNAs by RNA-sequencing. **b** Relative expression level of ACTA2-AS1 in CC and ANT tissues using RNA-sequencing. **c** The expression level of ACTA2-AS1 in CC tissues and ANT (n = 54) was measured by qRT-PCR. **d** The expression level of ACTA2-AS1 in human CC cell lines (Hela, SiHa and Caski) and human normal epithelial cell (HcerEpic) was investigated by qRT-PCR. ***P < 0.001, **P < 0.01, *P < 0.05

In our previous study, 241 differently expressed lncRNAs (fold change > 2.0 and P < 0.05) were identified between CC tissues and NCT by RNA sequencing (Fig. 1a). Among them, ACTA2-AS1 was significantly up-regulated by 22 times (Fig. 1b). Therefore, we further investigated the lncRNA ACTA2-AS1 expression level in 54 CC tissues and paired ANT by qRT-PCR. Results showed that ACTA2-AS1 levels were significantly increased in CC tissues compared with ANT (Fig. 1c). According to the median of the expression level of ACTA2-AS1, patients were classified into high-expression group (n = 28) and low-expression group (n = 26). Subsequently, the correlation between ACTA2-AS1 expression and clinical pathological characteristics of patients were also evaluated. The results (Table 1) showed that ACTA2-AS1 level was positively correlated with FIGO stage (P = 0.0293). However, ACTA2-AS1 expression was not significantly correlated with other clinicopathological factors such as age, tumor size, depth of invasion and lymphatic metastasis. Besides, we found that there was no correlation between ACTA2-AS1 and disease–free survival or overall survival in CC patients. Next, we examined expression levels of ACTA2-AS1 in normal cervical epithelial cells (HcerEpic) and CC cells (Caski, HeLa, and SiHa). As revealed in Fig. 1d, higher expressions were found in HeLa and SiHa cell lines compared to human normal epithelial cell. Thus, in subsequent studies, the specific function of ACTA2-AS1 in CC was further elucidated using the HeLa and SiHa cell lines as cell models. These data show that ACTA2-AS1 is strongly expressed in CC tissues and cells.

**Table 1:** Correlation analysis between ACTA2-AS1 expression and clinical features (n = 54)

Parameters	Total	ACTA2-AS1		*P*-value
		Low (n)	High (n)	
Age (years)				
< 50	26	14	12	0.4194
≥ 50	28	12	16	
Tumor size (cm)				
< 4	28	16	12	0.2712
≥ 4	26	10	16	
Depth of invasion				
< 2/3	25	14	11	0.2836
≥ 2/3	29	12	17	
FIGO stage				
0–I	27	17	10	0.0293*
IIa–IIb	27	9	18	
Lymphatic metastasis				
Yes	25	10	15	0.2659
No	29	16	13	

*P < 0.05

### Silencing of ACTA2-AS1 inhibited cell proliferation, colony formation and migration

**Fig. 2:**
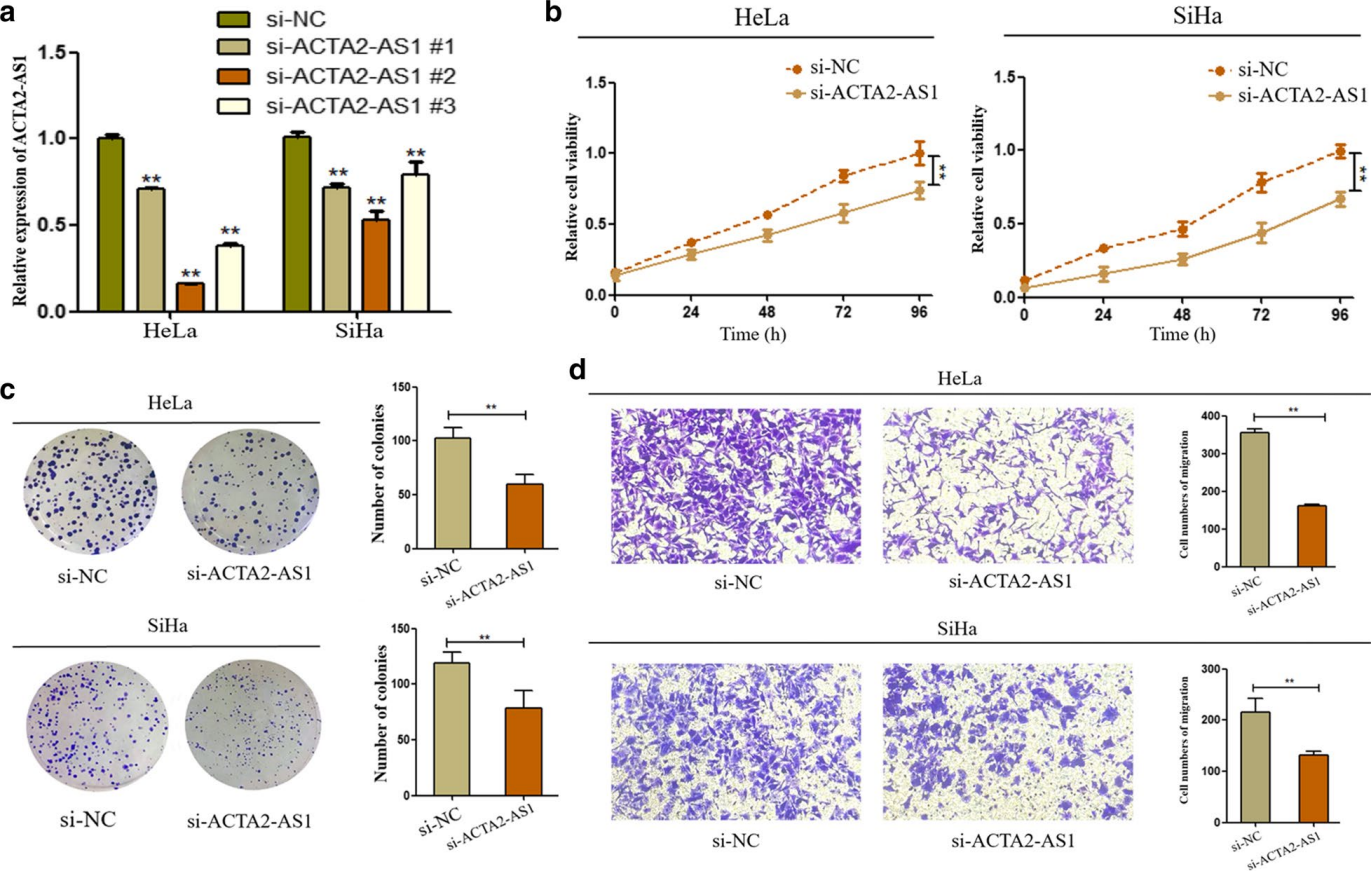
Silencing of LncRNA ACTA2‐AS1 inhibited proliferation, colony formation and migration of CC cells. **a** The knock-down ability of si-ACTA2-AS1 in HeLa and SiHa cells was confirmed by qRT-PCR. **b**, **c** CCK8 and colony formation assay revealed that the knock-down of ACTA2-AS1 could inhibit proliferation ability of CC cells. **d** Transwell assay manifested that silencing of ACTA2-AS1 could inhibit cell migration. The data are presented as mean ± SD. ** *P *< 0.01, NC: negative control

To further investigate the function of ACTA2-AS1 in CC progression, we constructed the siRNA of ACTA2-AS1 and transfected it into HeLa and SiHa cells to downregulate its expression. qRT-PCR results confirmed the efficiency 24 h after transfection. As presented in Fig. 2a, ACTA2-AS1 expression was reduced by three siRNAs. Of these, si-ACTA2-AS1#2 had the highest inhibition efficiency and was therefore selected for further functional studies. At first, CCK8 assay demonstrated that inhibition of ACTA2‐AS1 significantly suppressed the viability of SiHa and HeLa cells (Fig. 2b). Similarly, silencing of ACTA2‐AS1 could significantly inhibit the cell colony formation in HeLa and SiHa cells (Fig. 2c). Then, by transwell assay, down-regulation of ACTA2-AS1 was found to inhibit the migration of CC cells (Fig. 2d). These results identified that silencing of ACTA2-AS1 could inhibit cell proliferation, colony formation and migration capacity of CC cells.

### Effects of ACTA2-AS1 on cell apoptosis and cell cycle

**Fig. 3:**
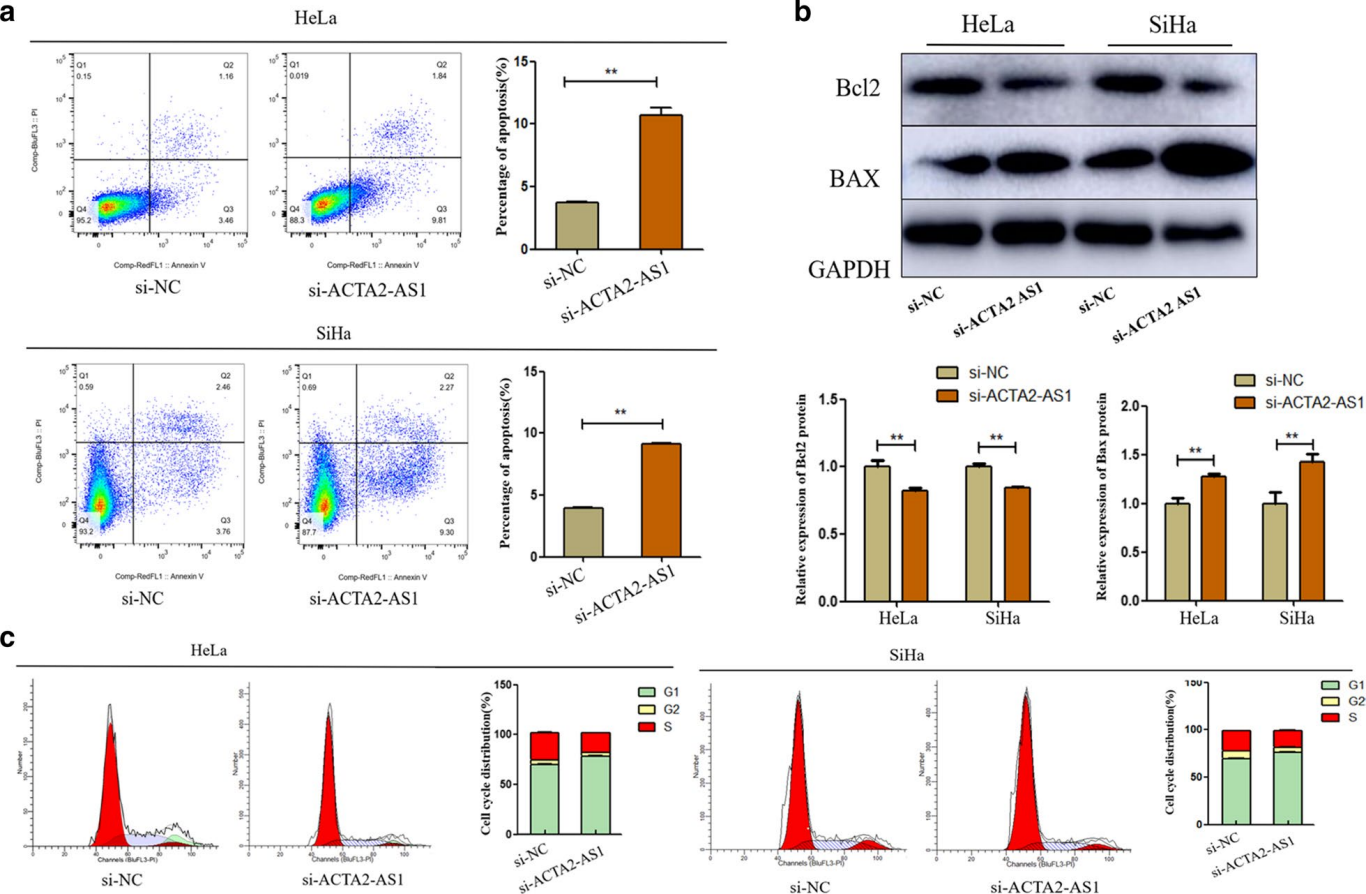
LncRNA ACTA2-AS1 influenced cell apoptosis and cell cycle. **a** Flow cytometry analysis was performed to detect the effects of ACTA2-AS1 on CC cell apoptosis. **b** Western blot assay investigates the changes of Bax and Bcl2 protein in ACTA2-AS1 silencing cells. **c** Flow cytometry assay was used to reveal the effects of ACTA2-AS1 on cell cycle. **P < 0.01

Further, we explored whether ACTA2-AS1 promoted cell proliferation by inducing apoptosis and regulating cell cycle. Apoptosis condition was assessed by flow cytometry analysis and western blot analysis of apoptosis-related proteins. As presented in Fig. 3a, downregulation of ACTA2-AS1 significantly promoted apoptosis in CC cells. Then, we investigated the expression of Bax and Bcl2 protein that are well-known for cell apoptosis by western blot assay. After ACTA2-AS1 was silenced, Bcl-2 protein expression was significantly decreased and Bax protein expression increased (Fig. 3b). This suggests that ATCA2-AS1 can affect apoptosis by affecting apoptosis-related regulator. In addition, cell cycle assay revealed that knock-down of ACTA2-AS1 resulted a G1 phase block to inhibit cell proliferation (Fig. 3c). The G1 phase of ACTA2-AS1-silencing HeLa cells (77.16 ± 0.49) was significantly higher than that of the NC group (69.1 ± 0.64) (P < 0.01). And the G1 phase of ACTA2-AS1-silencing SiHa cells (77.00 ± 0.81) was also significantly higher than NC group (69.1 ± 0.64) (P < 0.01). Based on these findings, we confirmed that ACTA2‐AS1 can affect cell proliferation and apoptosis in CC.

### ACTA2-AS1 served as a sponge of miR-143-3p

**Fig. 4:**
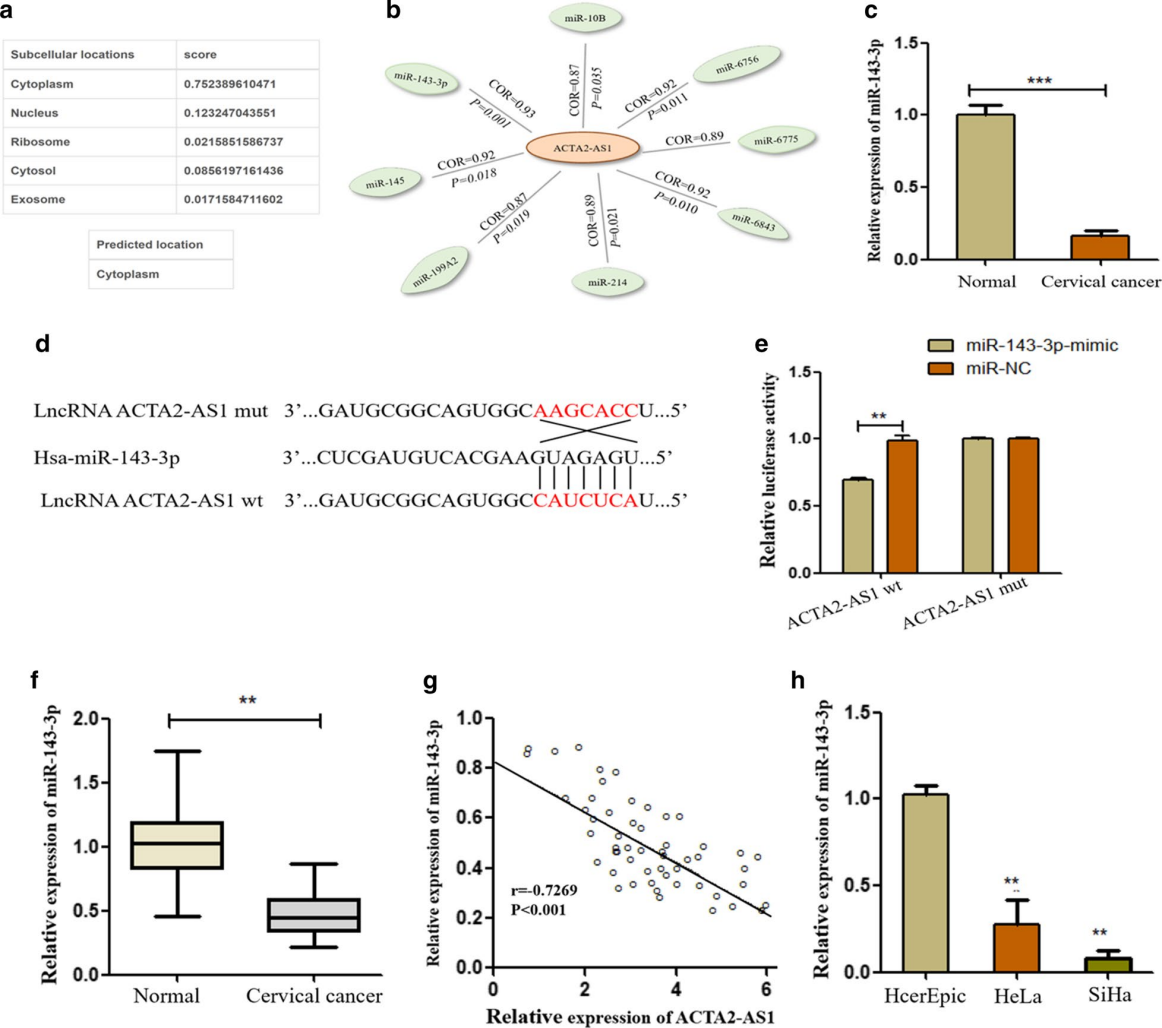
ACTA2-AS1 could serve as a sponge of miR-143-3p. **a** Prediction of subcellular localization of ACTA2-AS1 was performed. **b** miRNA co-expression analysis revealed a high correlation between miR-143-3p and ACTA2-AS1. **c** The expression of miR-143-3p in RNA sequencing results. **d** The binding sequence prediction of ACTA2-AS1-wt and construction of ACTA2-AS1-mut sequence. **e** Luciferase reporter assay demonstrated the miR-143-3p binding site in ACTA2-AS1. **f** The levels of miR-143-3p in CC tissues were tested. **g** Spearman’s correlation analysis analyzed the negative relation between ACTA2-AS1 and miR-143-3p in CC tissues. **h** Expression of miR-143-3p in cervical cancer cell lines was examined using qRT-PCR. ***P < 0.001, **P < 0.01

To further investigate the specific mechanism of ACTA2-AS1 in CC, we predicted its subcellular localization by lncLocator [21]. As shown in Fig. 4a, ACTA2-AS1 was found to be most likely to locate in the cytoplasm. Based on this, we hypothesized that ACTA2-AS1 primarily interacts with miRNA as a ceRNA and performed lncRNA-miRNA co-expression analysis. There are eight miRNAs potentially regulated by ACTA2-AS1, as shown in Fig. 4b, with the highest correlation coefficient for miR-143-3p. Furthermore, miR-143-3p was significantly down-regulated in our RNA sequencing results (Fig. 4c). We therefore hypothesized ACTA2-AS1 might act as a ceRNA of miR-143-3p and performed the next analysis. We found the presence of the miR-143-3p binding site on ACTA2-AS1 using DIANA-TarBase [22]. Subsequently, we designed ACTA2-AS1-wt and ACTA2-AS1-mut according to the binding site and conducted dual‐luciferase reporter assay (Fig. 4d). Results proved that miR‐143‐3p mimics could significantly reduce the luciferase activity of ACTA2‐AS1-wt, but not ACTA2‐AS1-mut (Fig. 4e). We then confirmed the expression levels of miR-143-3p in CC tissues and ANT by qRT-PCR. The results showed that miR-143-3p was weakly expressed in CC tissues compared to ANT (Fig. 4f). Besides, the negative relation between ACTA2-AS1 and miR-143-3p was demonstrated through Spearman’s correlation analysis (Fig. 4g). Similarly, miR-143-3p was also weakly expressed in CC cells compared to human normal epithelial cell (Fig. 4h). To further validate the correlation between ACTA2-AS1 and miR-143-3p, we investigated the expression level of miR-143-3p in ACTA2-AS1-silencing cells. The result manifested that miR‐143‐3p expression was efficiently elevated by silencing lncRNA ACTA2‐AS1 (Fig. S1a). To further upregulate and downregulate miR-143-3p, respectively, miR-143-p mimic/inhibitor and the matched NC were designed and transfected into HeLa and SiHa cells (Additional file 2: Fig. S1b, c). We then found that the expression of ACTA2-AS1 was suppressed after a significant increase in miR-143-3p expression (Additional file 2: Fig. S1d). Similarly, ACTA2-AS1 expression was elevated when miR-143-3p levels were inhibited (Additional file 2: Fig. S1e). Taken together, all these experimental results suggest that ACTA2-AS1 act as a ceRNA of miR-143-3p and they can inhibit reciprocally.

### ACTA2-AS1 modulated the expression of SMAD3 by repressing miR-143-3p

**Fig. 5:**
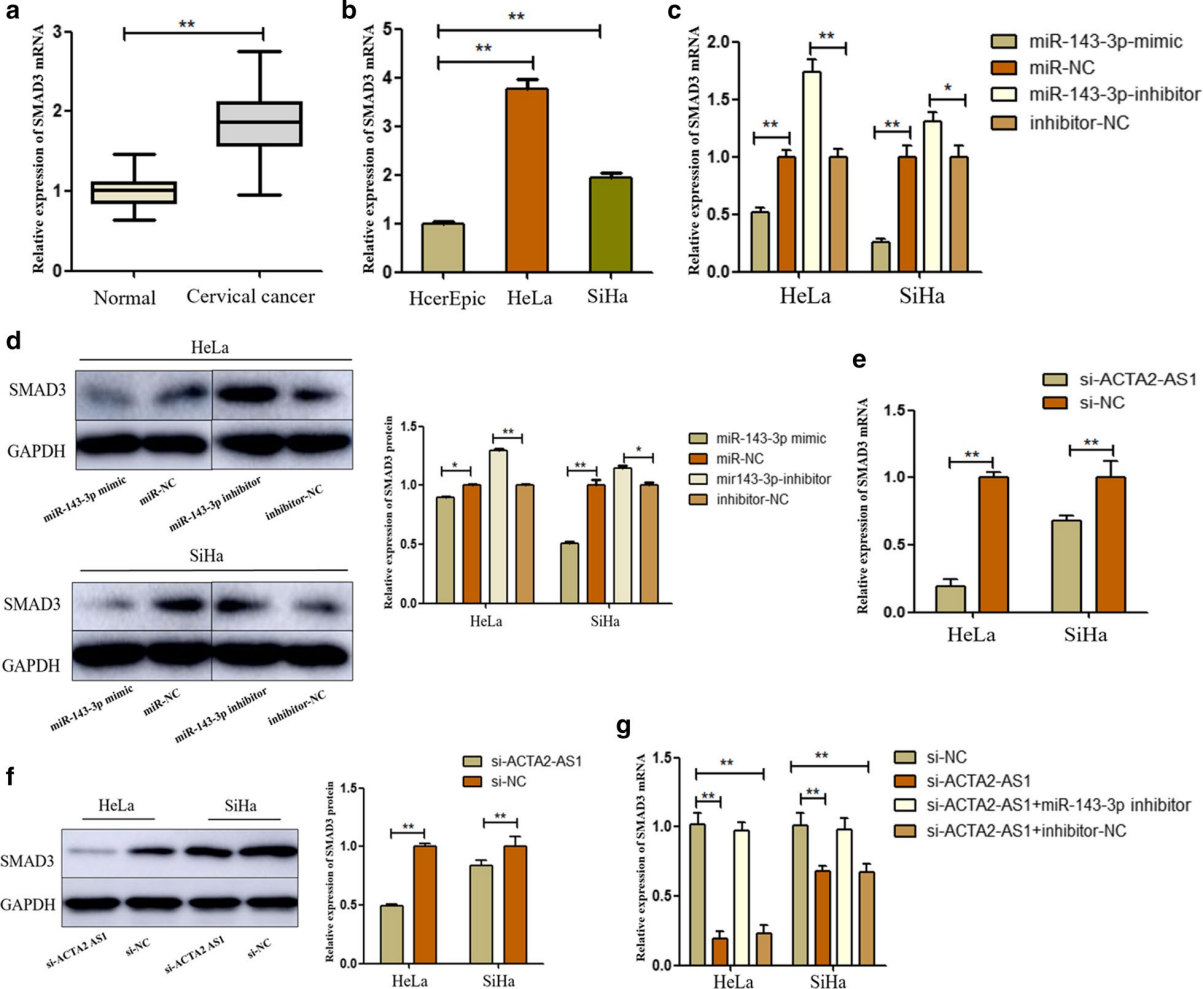
ACTA2-AS1 modulated the expression of SMAD3 by repressing miR-143-3p. **a** The mRNA levels of SMAD3 in CC tissues were tested using qRT-PCR. **b** The mRNA expression of SMAD3 in cervical cancer cell lines was examined. **c**, **d** The mRNA and protein expression levels of SMAD3 were determined after transfecting miR‐143‐3p mimic and inhibitor. **e**, **f**. The mRNA and protein expressions of SMAD3 were tested after silencing ACTA2‐AS1. **g** The mRNA expression of SMAD3 was detected after transfected with si-NC, si-ACTA2-AS1, si-ACTA2-AS1 + miR-143-3p inhibitor and si-ACTA2-AS1 + inhibitor NC, respectively. GAPDH was used as an internal control. *P < 0.05; ** *P *< 0.01

Since we found that ACTA2-AS1 acts as a ceRNA to regulate miR-143-3p, its downstream target genes need to be explored. To further investigate the potential target gene for miR-143-3p, we used Targetscan software (http://www.targetscan.org/vert_72/) [23] and found that SMAD3 is a potential target gene for miR-143-3p. Firstly, we verified the expression of SMAD3 in cervical cancer tissues and cell lines. As shown in Fig. 5a, SMAD3 was highly expressed in CC tissues compared to ANT. Similarly, the expression level of SMAD3 in CC cells was higher than that of human normal epithelial cells (Fig. 5b). Likewise, we further detected expression of SMAD3 after transfection with miR-143-3p mimic/inhibitor to prove the prediction. qRT-PCR manifested that miR‐143‐3p mimics suppressed the SMAD3 mRNA expression, while transfection of miR‐143‐3p inhibitors promoted the SMAD3 Mrna expression in HeLa and SiHa cells (Fig. 5c). Western blot assay also proved that protein expression of SMAD3 was regulated by miR-143-3p (Fig. 5d). Then, we investigated SMAD3 mRNA and protein expression in ACTA2-AS1-silencing cells. The results showed that down-regulation of ACTA2-AS1 could reduce the mRNA and protein expression of SMAD3(Fig. 5e, f). And the effect of si-ACTA2-AS1 on SMAD3 expression was attenuated by miR-143-3p inhibitor (Fig. 5g). In summary, above experiments proved that ACTA2-AS1 could regulate the expression of SMAD3 by sponging miR-143-3p in CC.

### ACTA2-AS1/miR-143-3p/SMAD3 could accelerate progression of CC

**Fig. 6:**
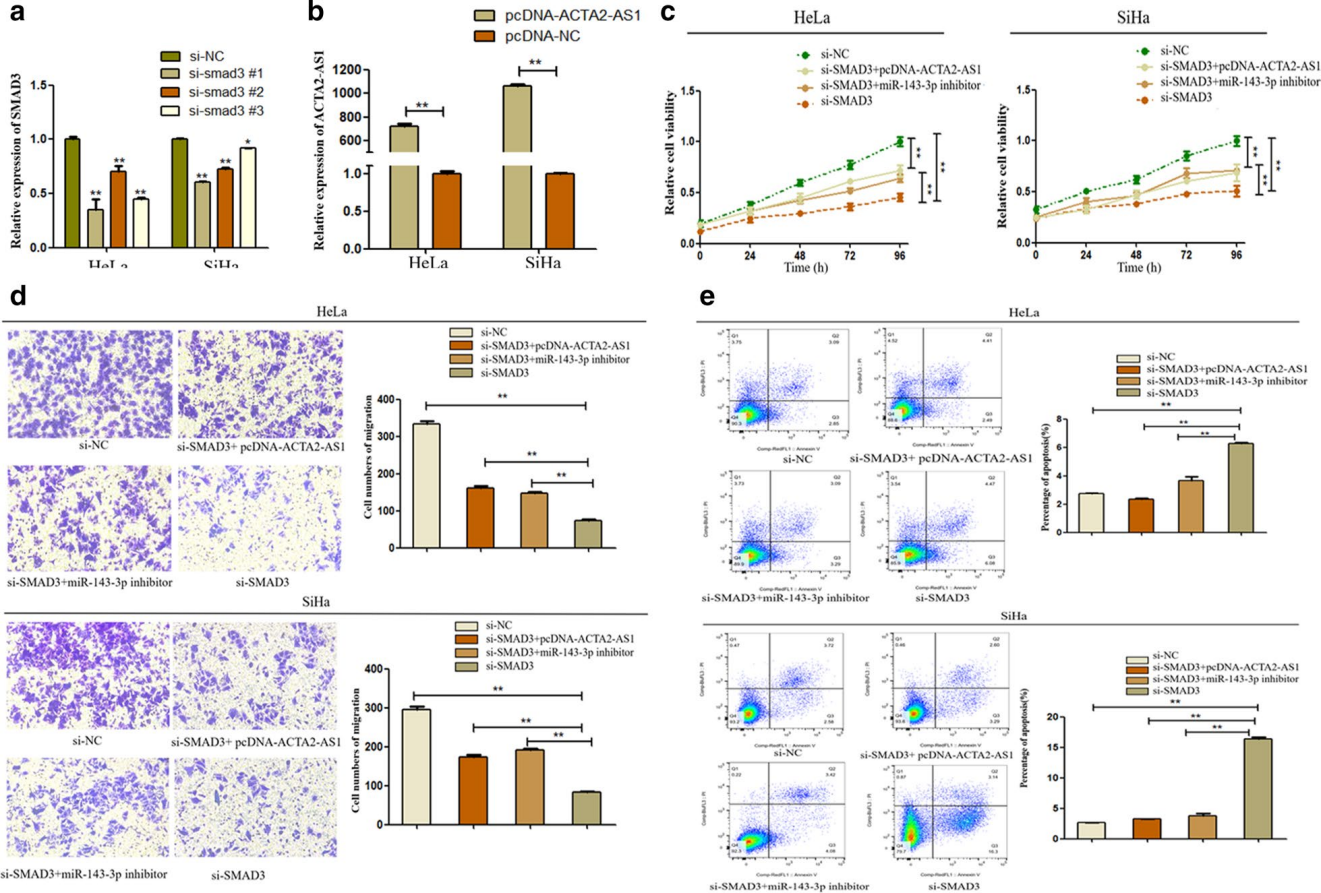
The effect of ACTA2-AS1/miR-143-3p/SMAD3 axis on progression of CC. **a** The knock-down ability of si-SMAD3 in HeLa and SiHa cells was confirmed by qRT-PCR. **b** The over-expression ability of pcDNA-ACTA2-AS1 was confirmed by qRT-PCR. **c** Cell viability was detected by CCK8 assay after transfecting with si-SMAD3 and co-transfecting with pcDNA‐ACTA2‐AS1 or miR-143-3p inhibitor. **d** The migration ability of transfected cells was analyzed by transwell assay. **e** Apoptosis condition of transfected cells was analyzed by flow cytometry analysis. ** *P* < 0.01, *P < 0.05

Based on the above results, we found that ACTA2-AS1 can act as a ceRNA to regulate miR-143-3p thus up-regulate SMAD3 expression. To strengthen our point, we further performed rescue assay to demonstrate the specific role of ACTA2-AS1/miR-143-3p/SMAD3 axis in CC progression. First, we designed and transfected SMAD3-specific siRNA into HeLa and SiHa cells. SMAD3 expression was reduced by three siRNAs and the si-SMAD3#1 with the best inhibition was selected for the next experiment (Fig. 6a). The pcDNA3.1 vector containing ACTA2-AS1(pcDNA-ACTA2-AS1) was then designed to upregulate ACTA2-AS1 expression and confirmed by qRT-PCR (Fig. 6b). We transfected si-SMAD3 into HeLa and SiHa cells simultaneously with pcDNA-ACTA2-AS1 or miR-143-3p inhibitor. According to the result of CCK8 assay, knock-down of SMAD3 significantly inhibited cell proliferation. Meanwhile, over-expression of ACTA2-AS1 or down-regulation of miR-143-3p partially reversed the inhibition effect of si-SMAD3 on proliferation compared with si-NC group (Fig. 6c). Then, transwell assay revealed that the inhibitory effect of si-SMAD3 on cell migration was also partially reversed again by co-transfection with pcDNA-ACTA2-AS1 or miR-143-3p inhibitor (Fig. 6d). Finally, down-regulation of SMAD3 notably promoted apoptosis in CC cells. As shown in Fig. 6e, the increased apoptosis rate resulting from si-SMAD3 was inhibited by overexpressing ACTA2-AS1 or silencing miR-143-3p. Taken together, we confirmed that silencing of SMAD3 inhibited cell proliferation, migration and promote apoptosis, which could be partially reversed by overexpression of ACTA2-AS1 or down-regulation of miR-143-3p. The ACTA2-AS1/miR-143-3p/SMAD3 axis exhibits an essential role in the development of cervical carcinogenesis.

## Discussion

Recently, increasingly studies have indicated that lncRNAs are abnormally expressed in human cancers and played an integral roles in progression of human cancers [24, 25]. LncRNA ACTA2‐AS1 was abnormally expressed in several human tumors and played a vital role in tumorigenesis. For example, in lung adenocarcinoma, high expression of ACTA2-AS1 is correlated with a relatively poor prognosis and could promote cell invasion and metastasis [12]. In hepatocellular carcinoma, ACTA2-AS1 was upregulated and promoted proliferation and metastasis by targeting α-SMA [13]. In breast cancer, ACTA2-AS1 showed a high potential in the prediction of survival but the specific role was unclear [14]. Nevertheless, the expression level and function role of ACTA2-AS1 in CC is still unclear. Our study firstly showed that ACTA2‐AS1 was highly expressed in malignant CC tissues compared to ANT. Clinically, the ACTA2-AS1 high expression group was correlated with a higher FIGO stage. Similarly, the expression level of ACTA2-AS1 was up-regulated in human CC cells, suggesting that ACTA2-AS1 may be involved in the CC progress.

Functionally, upregulated lncRNAs generally promote tumorigenesis by promoting cell proliferation, metastasis and inhibiting apoptosis [26, 27]. In our study, loss-of function assay proved that silencing of ACTA2-AS1 in CC significantly inhibited cell proliferation, migration, colony-forming capacity and caused G1 phase arrest. In addition, downregulation of ACTA2-AS1 significantly promotes apoptosis in CC. By detecting apoptosis-related proteins we speculated that down-regulation of ACTA2-AS1 may promote apoptosis through down-regulating the anti-apoptotic regulator Bcl2 [28] and up-regulating the pro-apoptotic regulator Bax [29]. These findings implicated that ACTA2‐AS1 exerts an oncogenic function and accelerates tumorigenesis in CC.

Investigating the mechanism of action of ACTA2-AS1 in CC is of great importance for understanding the occurrence, development and metastasis of CC. Subsequently, we further predicted the subcellular localization of ACTA2-AS1 primarily in the cytoplasm, suggesting that ACTA2-AS1 may regulate the progression of cervical cancer at the post-transcriptional level [5]. Mechanically, lncRNAs can regulate miRNA expression at the post-transcriptional level through competitive binding with miRNAs [15]. MicroRNAs are widely considered to be closely correlated with tumorigenesis by regulating the expression of their down-stream various oncogenes or tumor suppressor genes [30]. Through bioinformatics analysis, we found that miR-143-3p might be a downstream target of ACTA2-AS1. As a widely researched cancer related-miRNA, miR-143-3p has been verified to be lowly expressed in various cancers and could regulate cell proliferation, migration and invasion. For example, Sun et al. [31] proved that miR-143-3p was lowly expressed in osteosarcoma cancer tissues and cells, and inhibited cell proliferation, migration and invasion by targeting FOSL2. In Breast Cancer, Xia et al. [32] found that miR-143-3p was down-regulated in breast cancer cells and inhibited the cell proliferation and migration. Based on above findings, we hypothesized that the mechanism of ACTA2-AS1 involved in CC might be acting as a ceRNA of miR-143-3p. Luciferase reporter analysis convinced that miR-143-3p was a target gene of ACTA2 -AS1. Then, we proved that miR-143-3p was also downregulated in CC tissues and cell lines. Spearman’s correlation revealed a negative relation between ACTA2-AS1 and miR-143-3p in CC tissues. In addition, the expression of ACTA2-AS1 and miR-143-3p were reciprocally inhibited in CC cells. Collectively, these findings demonstrated that ACTA2-AS1 could target to miR-143-3p and they were negatively interacted.

Accumulating studies showed that lncRNAs can function as a ceRNA to regulate their target genes and then form a network in CC, such as OIP5-AS1/miR-448/Bcl-2 axis [33] and FOXP4-AS1/miR-136-5p/CBX4 axis [34]. Based on this, we supposed that ACTA2-AS1 might also regulate the target gene of miR-143-3p to exert its function. Furthermore, in a previous study, lncRNA-pathway network revealed that ACTA2-AS1 was associated with the Transforming growth factor‐β (TGF‐β) signaling pathway [35]. TGF‐β could promote tumor growth by triggering angiogenesis and mediating epithelial‐to‐mesenchymal transition in CC cells [36]. Therefore, we used Targetscan software to predict potential TGF‐β pathway related target of miR‐143‐3p and found a binding site between miR-143-3p and SMAD3. SMAD3, a member of the SMAD family, is reported to play an essential role in TGF-β1-mediated cancer progression [37]. Inhibition of Smad3 in the tumor microenvironment could suppress tumor growth, invasion and metastasis [37]. Thus, targeting SMAD3 and modifying the SMAD3-dependent tumor microenvironment may be an effective therapeutic approach against cervical cancer. In addition, SMAD3 can act as a target of multiple miRNAs to regulate tumor progression. For example, miR-17 could play as an oncogene by downregulating SMAD3 expression in hepatocellular carcinoma [38]. MiR-16-5p can inhibit the proliferation, invasion and metastasis of chordoma by targeting SMAD3 [39]. Therefore, we supposed ACTA2-AS1 might be participated in TGF‐β signal pathway through regulating SMAD3 expression via sponging miR-143-3p and form a network (Additional file 3: Fig. S2). In our study, we verified SMAD3 to be the target of miR-143-3p, which is consistent with Chen et al. [40]. Then, silencing of ACTA2-AS1 could reduce the expression of SMAD3. Finally, we conducted rescue assay to prove that overexpression of ACTA2-AS1 or inhibition of miR-143-3p could reverse the si-SMAD3 mediated inhibition on proliferation and migration of CC cells. Similarly, the effects resulting from si-SMAD3 on apoptosis was also inhibited through overexpressing ACTA2-AS1 or silencing miR-143-3p. However, whether ACTA2-AS1 can participate in the TGF-β pathway by regulating SMAD3 expression still requires further experimental demonstration. All the results showed that up-regulation of ACTA2-AS1 could promote SMAD3 expression via sponging miR-143-3p, formed a network.

## Conclusion

Taken together, our research found that ACTA2-AS1 was strongly expressed in CC tissues and was associated with a higher FIGO stage. Up-regulation of ACTA2-AS1 promoted the CC cell proliferation, migration and inhibited apoptosis. We found for the first time that ACTA2-AS1 can act as a ceRNA to regulate miR-143-3p expression and thus up-regulate its downstream SMAD3 expression. The ACTA2-AS1/miR‐43-3p/SMAD3 regulatory axis played an integral role in the pathogenesis and development of CC. Our findings will help to find a novel therapeutic target for CC and provide a basis for ACTA2-AS1 research mechanism.

### Supplementary information

**Additional file 1: Table S1.** Sequences of transfections.

**Additional file 2: Fig. S1** ACTA2-AS1 targeted miR-143-3p and was inhibited reciprocally. a The change of miR‐143‐3p expression was detected by qRT-PCR after silencing lncRNA ACTA2‐AS1. b, c. The expression of miR-143-3p was changed by miRNA mimic/inhibitor in HeLa and SiHa cells. d The expression level of ACTA2-AS1 was inhibited in miR-143-3p-upregulated CC cells. e ACTA2-AS1 expression was elevated when miR-143-3p levels were inhibited. **P < 0.01.

**Additional file 3: Fig. S2.** Schematic of the putative mechanism by which ACTA2-AS1 regulates the progression of cervical cancer.

## Data Availability

The datasets used and/or analyzed during the current study are available from the corresponding author on reasonable request.

## Abbreviations

CC: Cervical cancer
HPV: Human papillomavirus
LncRNAs: Long non-coding RNAs
miRNAs: MicroRNAs
ACTA2-AS1: ACTA2 antisense RNA1
ANT: Adjacent normal tissues
DMEM: Dulbecco’s Modified Eagle Medium
CCK8: Cell counting kit 8
NC: Negative control
siRNA: Small interfering RNA
SD: Standard deviation
ceRNA: Competitive endogenous RNA
TGF‐β: Transforming growth factor‐β
ANT: Adjacent normal tissues
ceRNA: Competitive endogenous RNA
qRT-PCR: Quantitative real-time PCR
