# Correction: A novel mechanism by which ACTA2-AS1 promotes cervical cancer progression: acting as a ceRNA of miR-143-3p to regulate SMAD3 expression

**DOI:** 10.1186/s12935-024-03243-2

**Published:** 2024-02-17

**Authors:** Lingli Luo, Min Wang, Xianping Li, Can Luo, Shan Tan, Sheng Yin, Lei Liu, Xiaolin Zhu

**Affiliations:** Department of Laboratory Medicine, The Second Xiangya Hospital, Central South University, Changsha, 410011 Hunan China

**Correction: Cancer Cell Int (2020) 20:372** 10.1186/s12935-020-01471-w

In this article [1], there was an error in Fig. 6. The corrected Fig. 6 is given below.

**Fig. 6:**
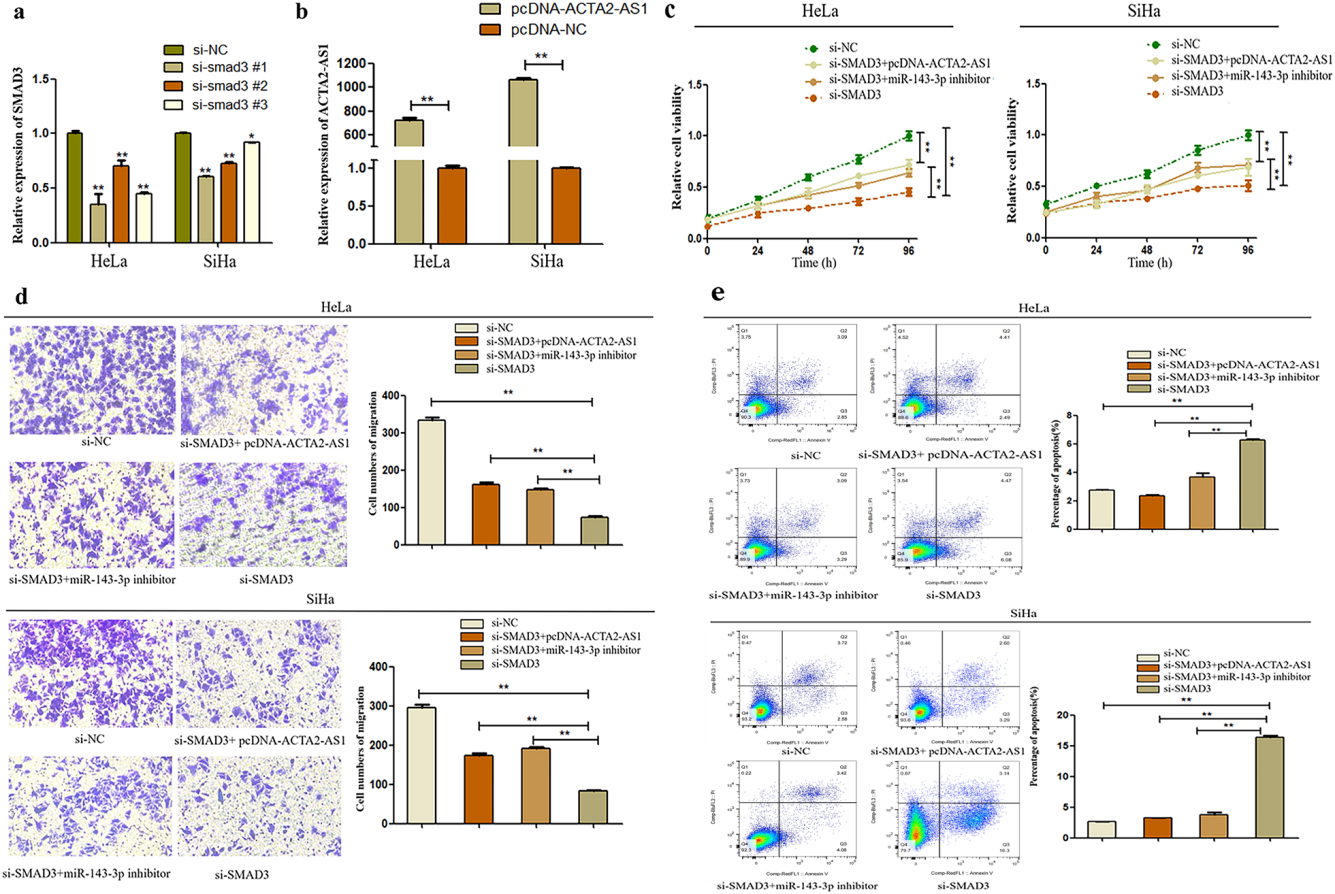
The efect of ACTA2-AS1/miR-143-3p/SMAD3 axis on progression of CC. **a** The knock-down ability of si-SMAD3 in HeLa and SiHa cells was confrmed by qRT-PCR. **b** The over-expression ability of pcDNA-ACTA2-AS1 was confrmed by qRT-PCR. **c** Cell viability was detected by CCK8 assay after transfecting with si-SMAD3 and co-transfecting with pcDNA‐ACTA2‐AS1 or miR-143-3p inhibitor. **d** The migration ability of transfected cells was analyzed by transwell assay. **e** Apoptosis condition of transfected cells was analyzed by fow cytometry analysis. ** P<0.05
