# Supplementary material for: A novel mechanism by which ACTA2-AS1 promotes cervical cancer progression: acting as a ceRNA of miR-143-3p to regulate SMAD3 expression
Source: Cancer Cell Int. 2020 Aug 5;20:372. doi: 10.1186/s12935-020-01471-w (PMC7409411; doi:10.1186/s12935-020-01471-w)
Supplement: Supplementary file 1 — Additional file 1: Table S1. Sequences of transfections. [file 12935_2020_1471_MOESM1_ESM.docx]

| Name | Sequences |
| --- | --- |
| si-ACTA2-AS1#1 | 5'-GAGGCUGGGAAGACAUAAUTT-3' |
| si-ACTA2-AS1#2 | 5'-GAGCCAUGGAUUUAGAAAUTT-3' |
| si-ACTA2-AS1#3 | 5'-CAUGCAUUGUCUGGGAAAUTT-3' |
| si-NC | 5'-UUCUCCGAACGUGUCACGUTT-3' |
| si-SMAD3#1 | 5'-GUGCUCCAUCUCCUACUACGATT-3' |
| si-SMAD3#2 | 5'-GGCUGCUCUCCAAUGUCAACATT-3' |
| si-SMAD3#3 | 5'-GCCUCAGUGACAGCGCUAUUUTT-3' |
| miR-143-3p mimic | sense 5'-UGAGAUGAAGCACUGUAGCUC-3' |
|  | antisense 5'-CAGCUACAGUGCUUCAUCUCA-3' |
| miR-NC | sense 5'-UUCUCCGAACGUGUCACGUTT-3' |
|  | antisense 5'-ACGUGACACGUUCGGAGAATT-3' |
| miR-143-3p inhibitor | 5'-GAGCUACAGUGCUUCAUCUCA-3' |
| anti-miR-NC | 5'-CAGUACUUUUGUGUAGUACAA-3' |

Table S1 Sequences of transfections
