# Supplementary figures and images for: A novel mechanism by which ACTA2-AS1 promotes cervical cancer progression: acting as a ceRNA of miR-143-3p to regulate SMAD3 expression
Source: Cancer Cell Int. 2020 Aug 5;20:372. doi: 10.1186/s12935-020-01471-w (PMC7409411; doi:10.1186/s12935-020-01471-w)

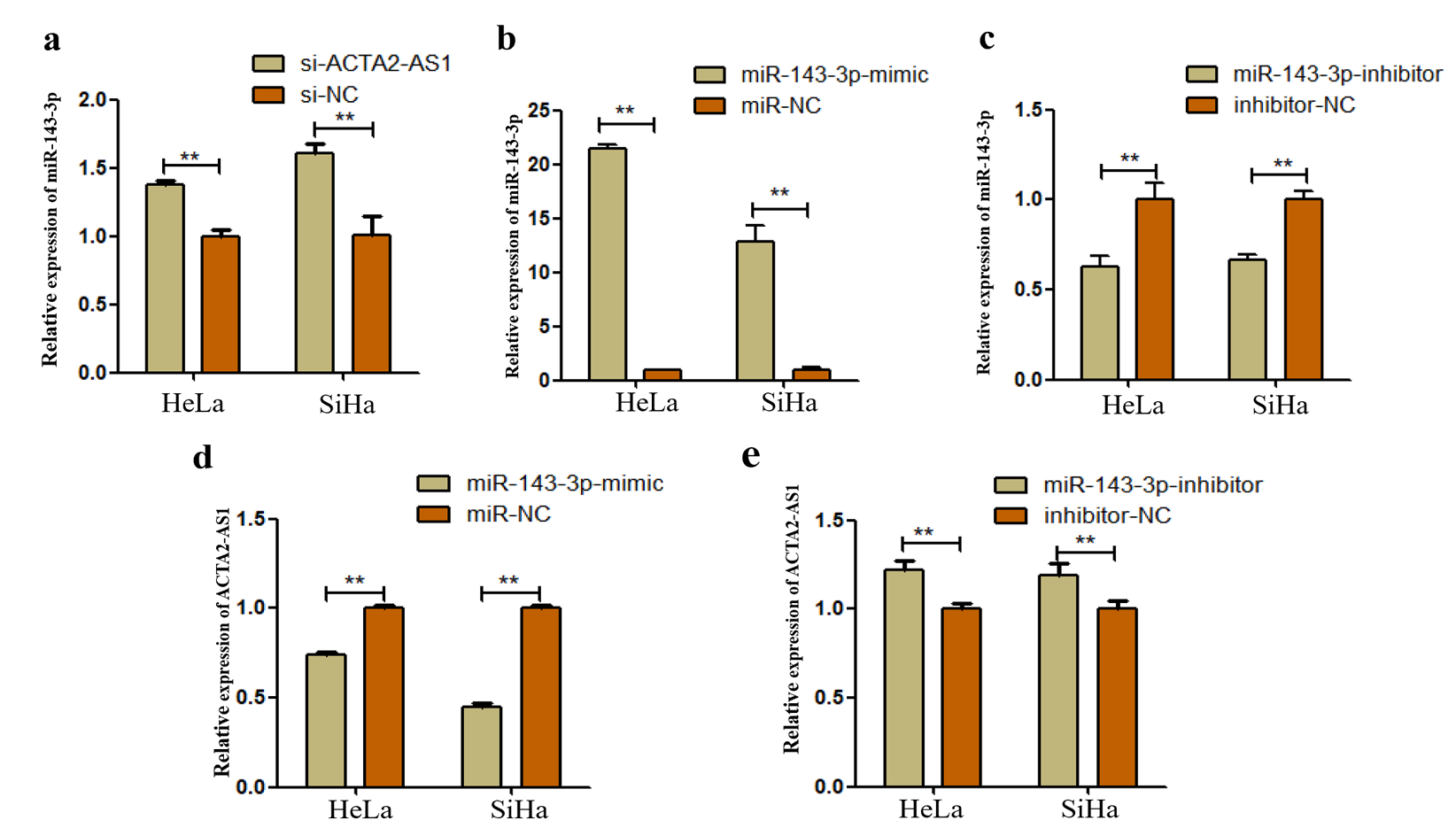

Supplement: Supplementary file 2 — Additional file 2: Fig. S1 ACTA2-AS1 targeted miR-143-3p and was inhibited reciprocally. a The change of miR‐143‐3p expression was detected by qRT-PCR after silencing lncRNA ACTA2‐AS1. b, c. The expression of miR-143-3p was changed by miRNA mimic/inhibitor in HeLa and SiHa cells. d The expression level of ACTA2-AS1 was inhibited in miR-143-3p-upregulated CC cells. e ACTA2-AS1 expression was elevated when miR-143-3p levels were inhibited. **P < 0.01. [file 12935_2020_1471_MOESM2_ESM.tif]

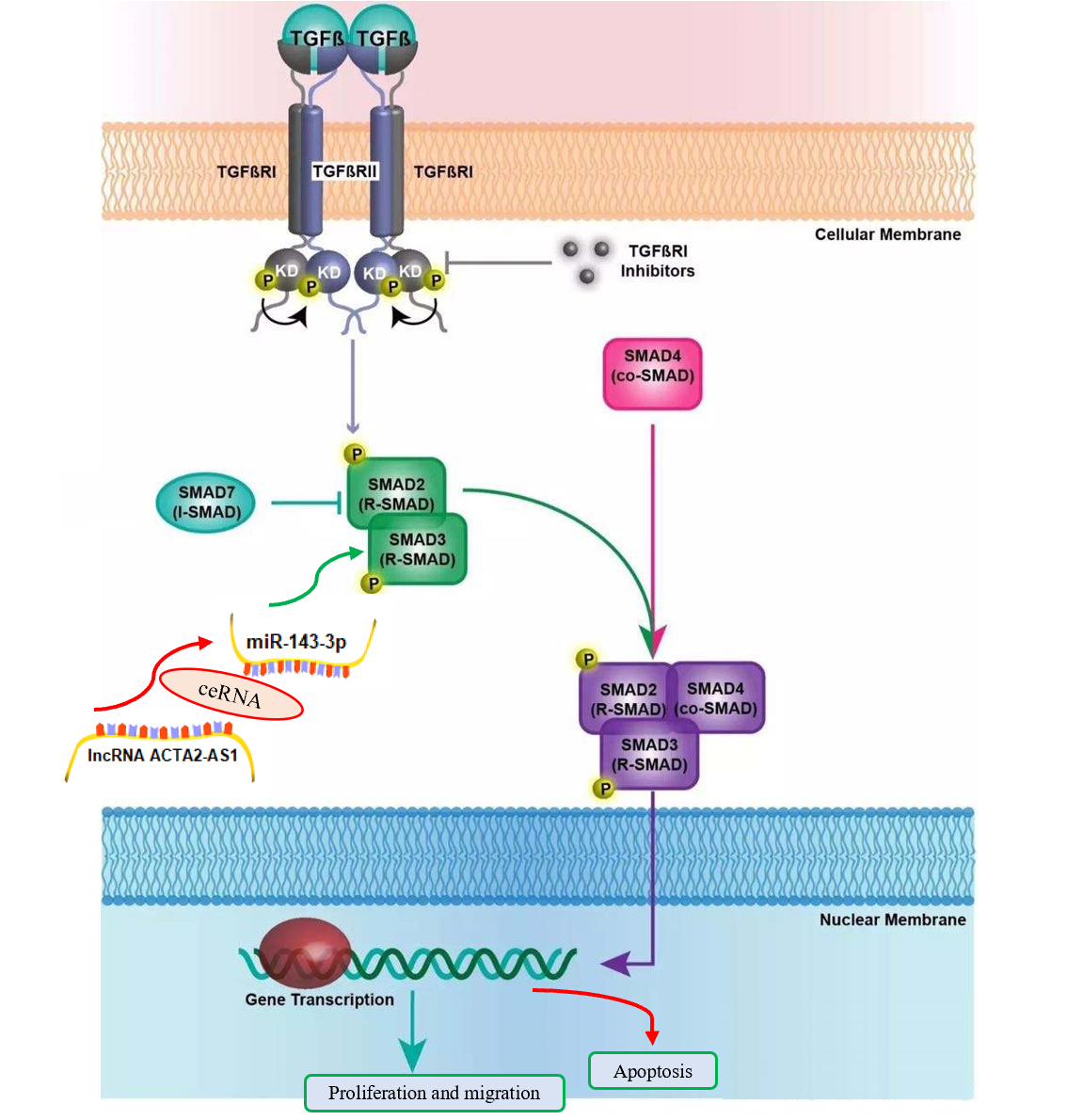

Supplement: Supplementary file 3 — Additional file 3: Fig. S2. Schematic of the putative mechanism by which ACTA2-AS1 regulates the progression of cervical cancer. [file 12935_2020_1471_MOESM3_ESM.tif]
